# White LED Light Exposure Inhibits the Development and Xanthophore Pigmentation of Zebrafish Embryo

**DOI:** 10.1038/s41598-019-47163-y

**Published:** 2019-07-25

**Authors:** Ünsal Veli Üstündağ, E. Çalıskan-Ak, Perihan Seda Ateş, İsmail Ünal, Gizem Eğilmezer, Türkan Yiğitbaşı, A. Ata Alturfan, Ebru Emekli-Alturfan

**Affiliations:** 1 Department of Biochemistry, Faculty of Medicine, Istanbul Medipol University, Kavacik, Istanbul Turkey; 2 Department of Histology and Embryology, Faculty of Dentistry, Marmara University, Maltepe, Istanbul Turkey; 3 Department of Biochemistry, Faculty of Dentistry, Marmara University, Maltepe, Istanbul Turkey; 4 Department of Biochemistry, Cerrahpasa Faculty of Medicine, Istanbul University-Cerrahpasa, Fatih, Istanbul Turkey

**Keywords:** Molecular medicine, Mechanisms of disease

## Abstract

Circadian rhythm in all living organisms is disturbed continuously by artificial light sources and artificial lighting has become a hazard for public health. Circadian rhythm of melatonin maintains high levels of melatonin during the night and low levels during the day. N-acetyltransferase (arylalkylamine N-acetyltransferase, AANAT) is one of the four enzymes required for melatonin synthesis and *mtnr1ba* is a melatonin receptor-encoding mRNA that is expressed widely in the embryonic brain. Pax7 has important roles during neural crest development and especially xanthophore pigmentation. Due to its diurnal nature, zebrafish provide a special opportunity for research on circadian rhythms that are regulated by melatonin. Here in this study, we showed that when compared with the white light control group, white LED light exposure resulted in loss of yellow pigmentation, decreased body length and locomotor activity, oxidant-antioxidant imbalance and decreased expressions of *aanat2*, *mtnr1ba*, and *pax7* in zebrafish embryos. Histological analysis of this group revealed disorganization of the spaces among photoreceptor cells, decreased total retinal thickness and photoreceptor cell layer thickness compared with the control group. Artificial lighting pollution has the potential to become an important risk factor for different diseases including cancer especially for industrialized countries, therefore, more studies should be performed and necessary regulations should be made regarding this risk factor.

## Introduction

Sunlight is a major factor in the maintenance of the biological clock and the regulation of the circadian rhythm in all living organisms. On the other hand, this natural process is disturbed continuously by artificial light sources that include various electromagnetic spectrums and technological tools. Artificial lighting has become an important component of urban life. On the other hand, artificial light exposure has complicated the management of the biologic metabolic processes synchronously^1–4^.

Photoperiod is the main signal for the regulation of endogenous rhythms. Suprachiasmatic nucleus (SCN) activation is effective in locomotive activity, water and nutrient intake, sexual behavior, body temperature, ACTH release, prolactin and melatonin release^1–3^. Circadian rhythm of melatonin circulation is an important characteristic of vertebrate physiology to maintain high melatonin levels during night time and low levels during day time^5,6^. During night melatonin is produced at pineal gland and retinal photoreceptor cells. In the pineal gland circulating melatonin is produced which acts as a hormone in seasonal and circadian physiology and the melatonin produced in retinal photoreceptor cells is suggested to regulate light and darkness adaptation. Tryptophan is the precursor of melatonin and serotonin N-acetyltransferase (arylalkylamine N-acetyltransferase, AANAT) is one of the four enzymes required for melatonin synthesis from tryptophan^5–7^. Melatonin levels in circulation are controlled mainly by AANAT activity. On the other hand, external light signals have been reported to control the daily rhythm in AANAT activity^7^. Melatonin is produced in the adult pineal gland of zebrafish regulated by a pineal circadian clock upon the action of light on pineal photoreceptors^8–10^. The mammalian melatonin receptor MT1 is found in the brain, whereas MT2 which is homologous to zebrafish *mtnr1ba* is localized to the retina^11,12^. In zebrafish melatonin receptors are distributed as *mtnr1aa*, *mtnr1ba*, and *mtnr1bb*, that are expressed widely in the embryonic brain^13^. However, in the adult zebrafish, *mtnr1ba* and *mtnr1bb* are expressed mainly in the periventricular gray zone of the optic tectum and in the periventricular thalamus and hypothalamus^14^.

Three types of chromatophores as black melanophores, yellow xanthophores, and shimmering iridophores form the characteristic golden and blue horizontal stripes of zebrafish. These chromatophores are originated from neural crest cells in the embryo/early larvae^15^. In fish and reptiles, the effects of melatonin differ depending on the species, the developmental stage, and the location of the pigment cell. The paired box homeodomain transcription factor Pax7 has important roles during neural crest development and especially xanthophore pigmentation^16^.

Light emitting diodes (LED) are used as alternative and effective light sources, they are cost efficient and they have a broad beam width^11^. Some countries have forced the use of fluorescent bulbs or LEDs instead of incandescent bulbs to save energy. On the other hand, LEDs emit a wavelength of light which is related with detrimental effects on human health^12^. Accordingly, in recent years, it has been recognized that artificial lighting has health implications^4^. Artificial light exposure has been related to diabetes, different types of cancer and depression. Recent studies have shown that artificial light exposure affects cell differentiation, growth, and development and cause cardiovascular diseases, diabetes, obesity, neurological diseases due to changes in the endocrine system as well as different types of cancer^4,13–20^.

Zebrafish have become a popular model organism due to its external fertilization, small size, high reproductivity, rapid development, transparency, suitability for screening and rapidly growing scientific databases^21^. Unlike nocturnal rodents, zebrafish are generally regarded as diurnal organisms that are active during daytime in both adult and larval stages^16^. Diurnal feature of zebrafish makes them special organisms for circadian rhythm research regulated by melatonin. However, the role of the light spectrum has not been extensively investigated in zebrafish.

Although people, especially children, are exposed to LED lights in daylight due to electronic equipment, to our knowledge, there is no information about the daylight effects of different LED lights on melatonin-expressing gene (*aanat* 2), locomotor activity, oxidant-antioxidant status in zebrafish embryos. Moreover, we hypothesized that LED light exposure might affect the melatonin receptor-expressing gene *mtnr1ba* which is localized in the retina in zebrafish, for that reason we measured *mtnr1ba* expression and examined the retina histologically. During our experiments, we observed that white LED light exposure reduced the xanthophore pigmentation. To investigate the underlying mechanism, we also examined the expression of the *pax7* gene responsible for xanthophore pigmentation.

## Results

**Figure 1:**
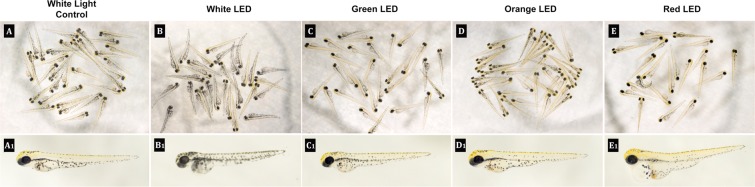
Representative images of the zebrafish embryos at 72 hpf. Examples of individual phenotypes are given in A1–E1.

Representative images of the zebrafish embryos at 72 hpf are given in Fig. 1. Examples of individual phenotypes are given in A1-E1. Morphological abnormalities such as pericardial edema were observed in red LED exposed zebrafish (Figure D1). Loss of yellow pigmentation was apparent in the white LED exposed group (Figure E1).

### Quantification of pigmentation

**Figure 2:**
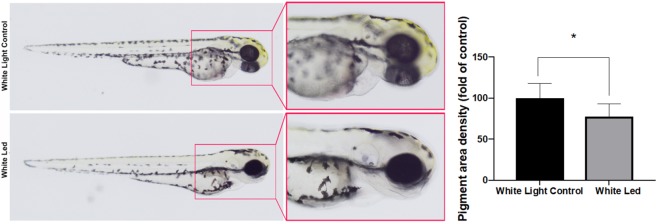
Effects of White LED light pigmentation in zebrafish embryos. (A) Synchronized embryos (n = 20) were exposed to White LED light and White light control. The effects on zebrafish pigmentation were observed under a stereomicroscope at 3 dpf. The pigmentation area density in the treated embryos was normalized to that of the control embryos using the ImageJ software. *p < 0.05, compared to the control.

The treatment of the zebrafish embryos with white LED light significantly decreased yellow pigmentation in the developing larvae (Fig. 2). The density of the pigmented area in the treated embryos was normalized to that in the control embryos using Image J software.

### Mortality and hatching rate results

**Figure 3:**
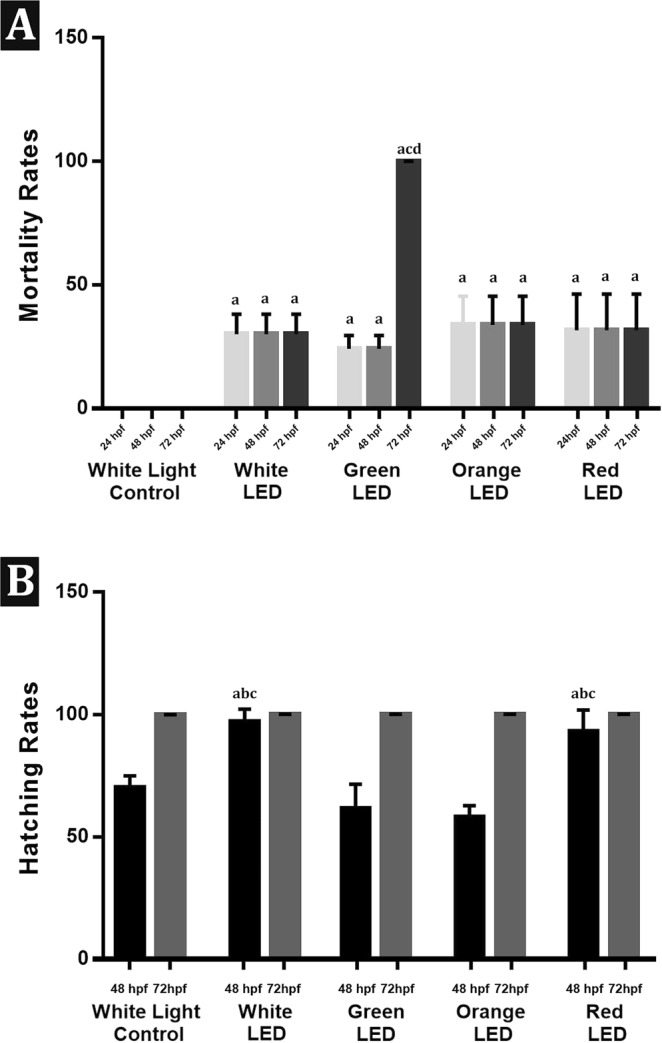
(**A**) Mortality rates of the embryos in the white light control and experimental groups; Values are given as mean ± standart deviation. ^a^p < 0,05; Significantly different from the respective control, ^c^p < 0,05; Significantly different from Orange-72 hpf, ^d^p < 0,05; Significantly different from Red-72 hpf, ^e^p < 0,05; Significantly different from White-72 hpf. (**B**) Hatching rates of the embryos in the control and experimental groups, ^a^p < 0,05; Significantly different from the respective control ^b^p < 0,05; Significantly different from Green-48 hpf, ^c^p < 0,05; Significantly different from Orange-48 hpf.

Mortality rates and hatching rates of the embryos in the white light control and experimental groups are given in Fig. 3A,B. Mortality rates increased significantly in all light exposed groups when compared with their respective controls. On the other hand, the mortality rate of the blue LED exposed group at 72 hpf was significantly higher than all other groups. At 48 hpf hatching rate of the white LED exposed group was significantly higher than the white light control, green and orange LED exposed groups.

### Body length and locomotor activity

**Figure 4:**
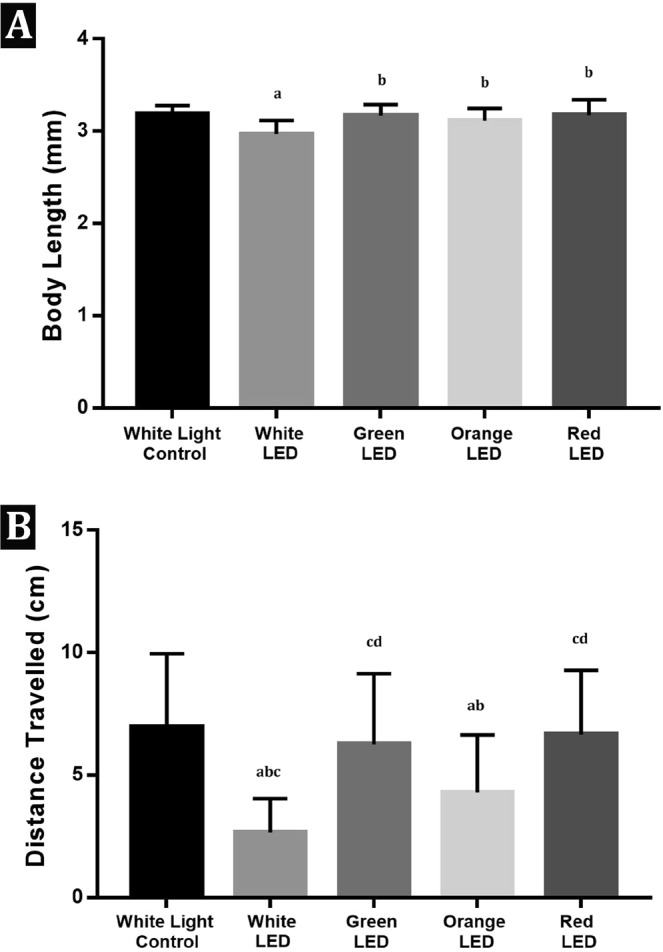
(**A**) Body length of the embryos in the white light control and exposure groups. Values are given as mean ± standart deviation. ^a^p < 0,05; significantly different from the white light control group; ^b^ significantly different from the White LED Group. (**B**) Total distance travelled (cm) by the embryos in the white light control and exposure groups ^a^p < 0,05; significantly different from the white light control Group; ^b^p < 0,05; significantly different from Green LED Group; ^c^p < 0,05; significantly different from Orange LED Group; ^d^p < 0,05; Significantly different from White LED Group.

Body lengths of the embryos in the white light control (3,19 ± 0,09 mm) and white (2,98 ± 0,15 mm), green (3,17 ± 0,12), orange (3,11 ± 0,13) and red (3,17 ± 0,17) LED groups are given in Fig. 4A. The body length of the white LED exposed group decreased significantly when compared with the white light control and the other groups (Fig. 4A). Results of the behavioral assay indicated as the total distance swam is given in Fig. 4B. There was a strong inhibition in the locomotor activity of the white LED exposed embryos (2,66 ± 1,38 cm) when compared with the white light control (6,96 ± 3,0 cm) and other exposure groups (6,26 ± 2,88 cm; 4,30 ± 2,34 cm; 6,66 ± 2,62 cm for green, orange and red LED groups respectively).

### Biochemical assay results

**Figure 5:**
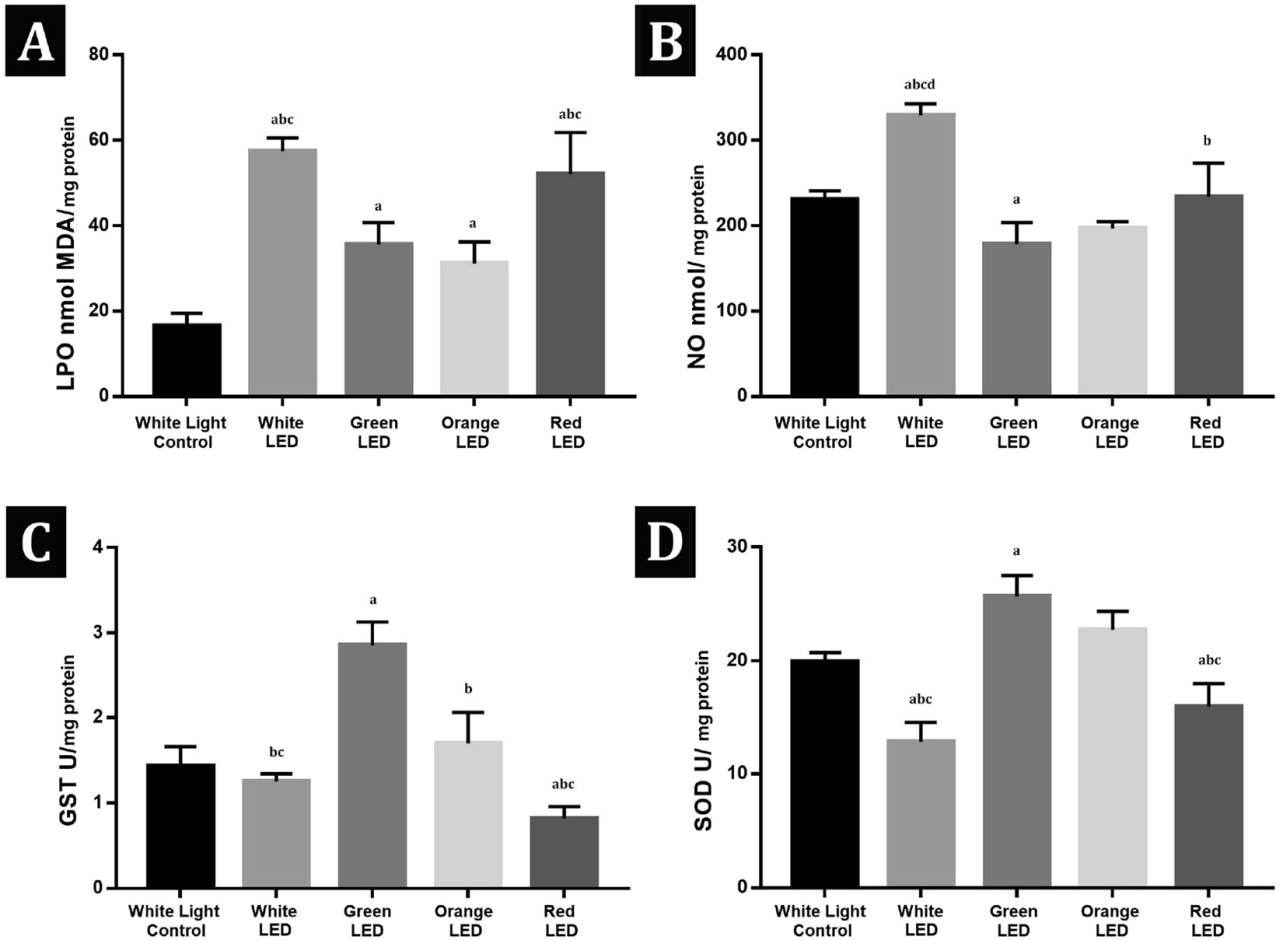
(**A**) Lipid peroxidation (LPO) levels (nmol MDA/gr) of the white light control and the exposure groups. (**B**) Nitric oxide (NO) levels (nmol NO/mg protein) of the white light control and the exposure groups. **(C)** Glutathione S-transferase (GST) activities (U/mg protein) of the white light control and the exposure groups **(D)** Superoxide dismutase (SOD) activities (U/mg protein) of the white light control and the exposure groups. Values are given as mean ± standart deviation. ^a^p < 0,05 Significantly different from the Control Group; ^b^p < 0,05 Significantly different from Green LED Group; ^c^p < 0,05 Significantly different from Orange LED Group; ^d^p < 0,05 Significantly different from Red LED Group.

Spectrophotometric analysis showed significant increases in LPO levels in all light exposed groups when compared with the white light control group. Red and white LED light exposed groups had significantly higher LPO levels (52,13 ± 9,7 nmol MDA/mg protein; 57,46 ± 3,08 nmol MDA/mg protein respectively) than the green and orange LED light exposed groups (35,47 ± 5,3 nmol MDA/mg protein; 31,07 ± 5,07 nmol MDA/mg protein respectively). NO levels significantly increased in the white LED exposed group (329,4 ± 13,58 nmol/mg protein) when compared with the white light control (230,9 ± 9,77 nmol/mg protein) and the other exposure groups but decreased in the green LED light exposed group (178,3 ± 25,47 nmol/mg protein) when compared with the white light control group. SOD and GST activities significantly increased in the green LED light exposed group (25,67 ± 1,85 U/mg protein ; 2,86 ± 0,27 U/mg protein respectively) and decreased in the red LED light exposed group (15,92 ± 2,01 U/mg protein; 0,82 ± 0,14 U/mg protein respectively) when compared with the white light control group (19,94 ± 0,8 U/mg protein; 1,43 ± 0,23 U/mg protein). Red and white LED light exposed groups had significantly decreased SOD and GST activities (12,84 ± 1,7 U/mg protein; 1,25 ± 0,09 U/mg protein White LED group respectively) when compared with the green and orange LED light exposed groups (22,73 ± 1,64 U/mg protein; 1,7 ± 0,36 U/mg protein Orange LED group respectively) (Fig. 5).

### RT-PCR analysis results

**Figure 6:**
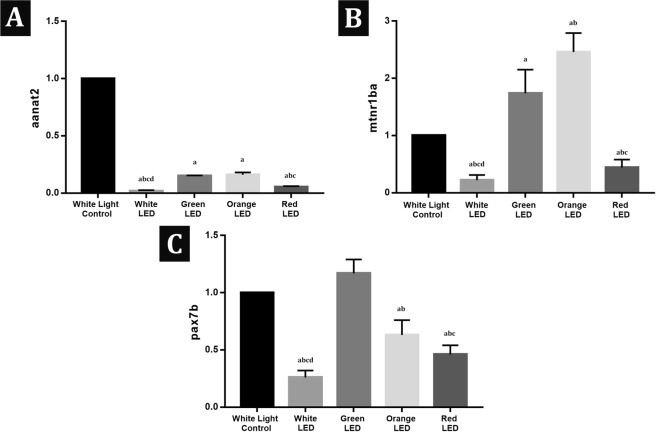
(**A–C**) Bar graph presentation of the fold change of *aanat2*, *mtnr1ba* and *pax7b* transcripts quantified by RT-PCR. All RT-PCR results are normalized to β-actin, the housekeeping gene and expressed as change from their respective controls. Data presented are mean ± SD. Significant difference is indicated by asterisk ^a^p < 0,05 Significantly different from the white light control group; ^b^p < 0,05 Significantly different from the Green LED Group; ^c^p < 0,05 Significantly different from the Orange LED Group; ^d^p < 0,05 Significantly different from the Red LED Group.

The expressions of *aanat2*, *mtnr1ba* and *pax7b* are given as fold change of transcript quantified by RT-PCR. RT-PCR results were normalized to β- actin, housekeeping gene, and expressed as change from their respective controls. The average values obtained from the three experiments are given in Fig. 6. *aanat2* expression decreased significantly in all exposure groups when compared with the white light control group. Green and orange LED exposure increased (1,74 ± 0,41; 2,46 ± 0,33 respectively) whereas red and white LED exposure decreased (0,44 ± 0,14; 0,22 ± 0,09 respectively) *mtnr1ba* activity significantly. *pax7b* activities decreased significantly in the orange (0,63 ± 0,13), red (0,46 ± 0,08) and white LED (0,26 ± 0,06) groups compared with the white light control group. White LED exposed group had the lowest *aanat2* (0,02 ± 0,01), *mtnr1ba* (0,22 ± 0,09) and *pax7b* (0,26 ± 0,06) expressions among the groups.

### Histological evaluations results

**Figure 7:**
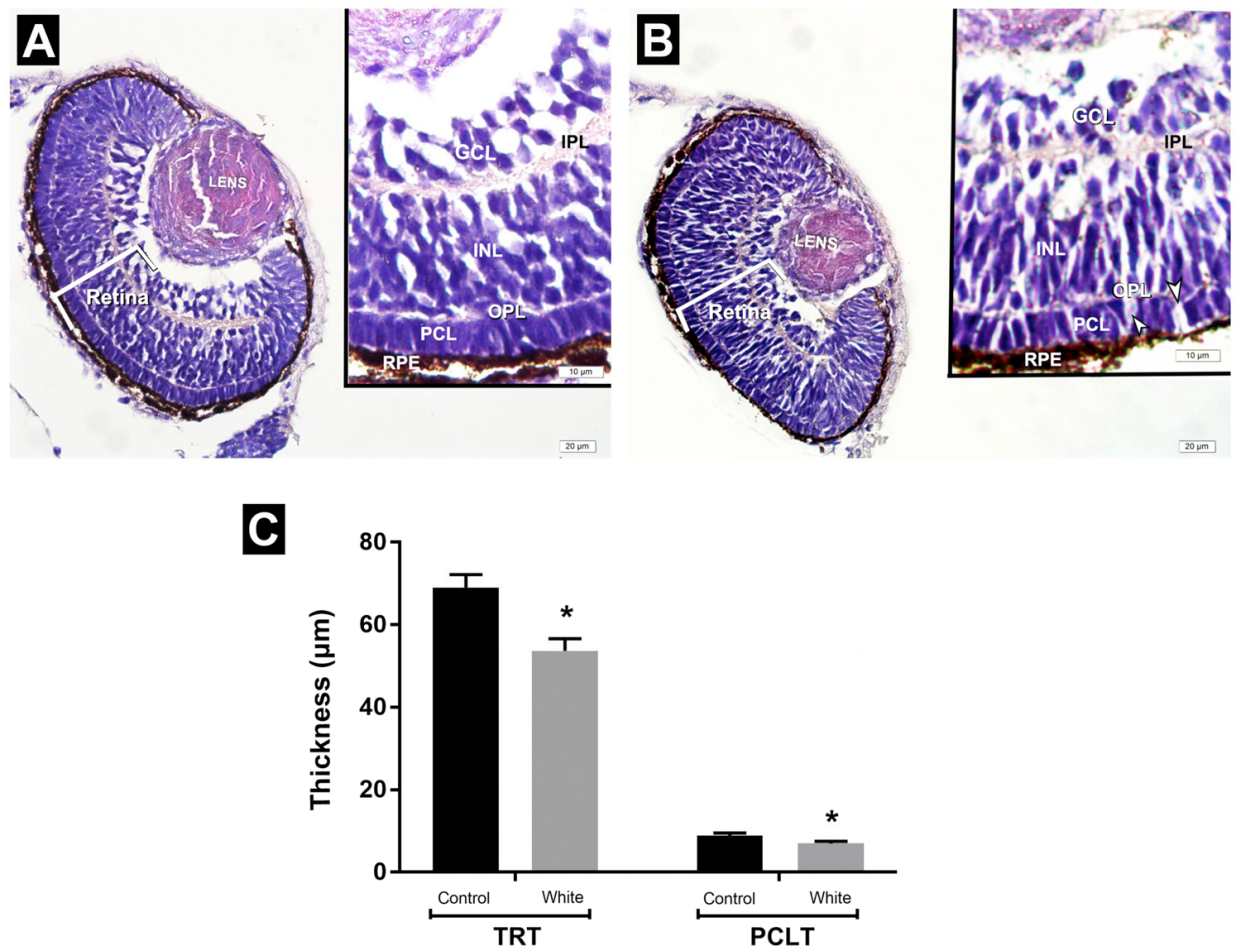
(**A**,**B**) Representative photomicrographs of the eyes of zebrafish embryos in the white light control and white LED light exposed groups at 72 hpf. White light control group. (**A**) Retina with regular layers. White LED light group. (**B**) disorganization of photoreceptor cells including spaces among cells. (**C**) Assesment of measurement of the thickness of total retina and photoreceptor cell layer. INL: Inner nuclear layer; GCL: Ganglion cell layer; PCL: Photoreceptor cell layer; IPL: Inner plexiform layer; OPL: Outer plexiform layer; TRT: Total retinal thickness; PCLT: Photoreceptor cell layer thickness; HE staining.

Light microscopic investigation of white light control group exhibited retina structure with regular layers. After 3 days of white LED light treatment, photoreceptor cells revealed disorganization of the spaces among photoreceptor cells. Measurement of the retina demonstrated that total retinal thickness significantly decreased in the white LED light group (53,7 ± 2,9 μm) compared to the white light control group (69,5 ± 3,13 μm). Also, the thickness of the photoreceptor cell layer in the white LED light group (6,9 ± 0,52 μm) was significantly lower than the white light control group (8,9 ± 0,57 μm) (Fig. 7).

### *In situ* hybridization results

**Figure 8:**
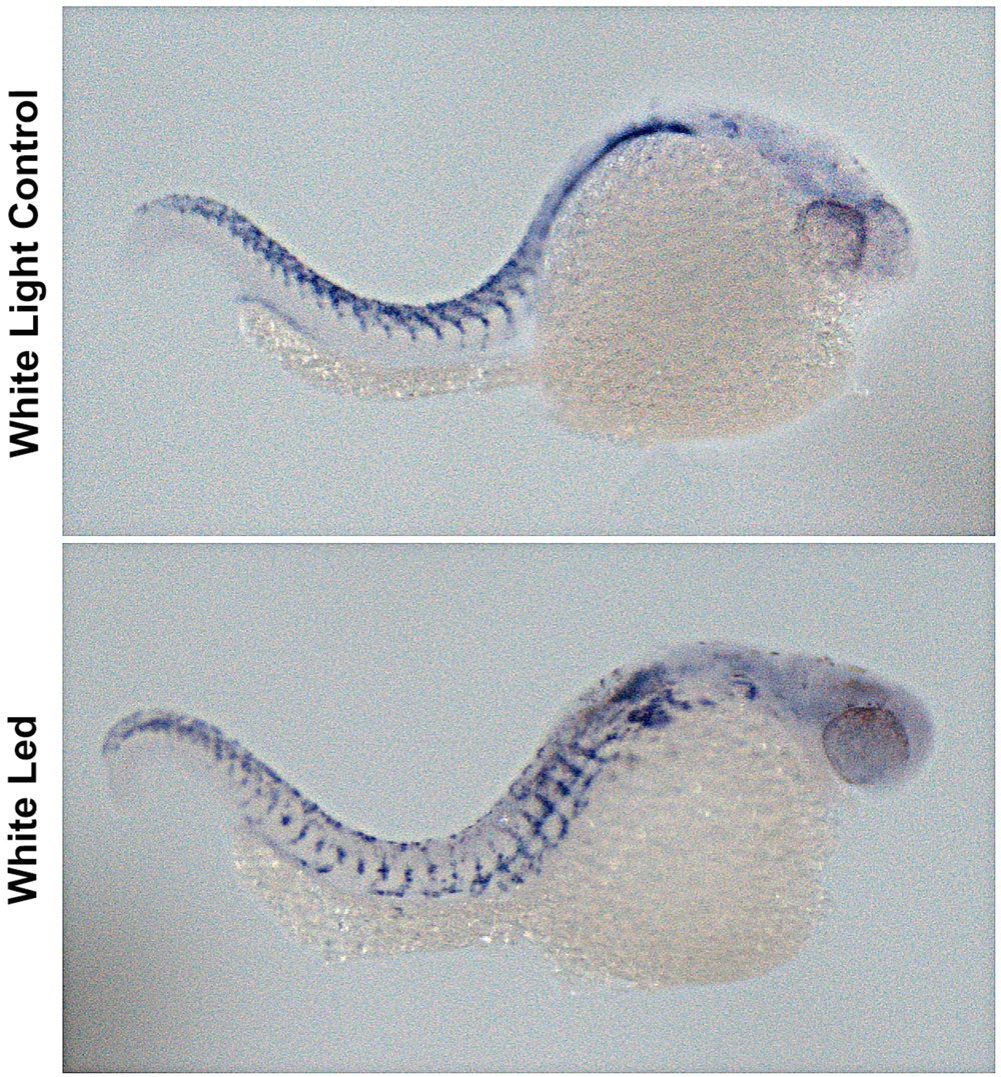
*In situ* hybridization results revealed decreased crestin expression in white LED exposed group when compared with the white light control group.

*In situ* hybridization results revealed decreased crestin expression in white LED exposed group when compared with the white light control group (Fig. 8).

## Discussion

The results of our study show that artificial lighting using LEDs affected the morphology, mortality and hatching rates, locomotor activity, oxidant and antioxidant parameters and gene expressions related with melatonin circulation, *mtnr1ba*, and *aanat2*, as well as *pax7* which is an important regulator of xanthophore production in zebrafish embryos.

Under dark conditions melatonin which is a neuro-hormone generated and secreted by the pineal gland and regulates many important body functions. Light is the regulator of melatonin production and even light with low intensity and short duration have been shown to suppress melatonin production^22–24^. Nocturnal pineal melatonin generation is very photosensitive to the light with short wavelength such as blue light^25,26^.

Zebrafish is a diurnal animal and an attractive model organism to investigate the circadian rhythm as they have conserved clock mechanisms shared with mammals^27^. In zebrafish, melatonin is generated both in the pineal gland and in the retina. Retina has been suggested to regulate the pineal melatonin secretion and also produce its own melatonin independent of the pineal gland. Although retinal and pineal melatonin is produced during dark in most vertebrates, the process is different in zebrafish. Zebrafish retinal melatonin production depends on different retinal cell types, such as photoreceptors, interneurons, and ganglion cells all expressing the Aanat 2 enzyme that catalyzes the transformation of serotonin into melatonin^28^.

Accordingly, our study was designed to evaluate the effects of light with different wavelengths on the expression of enzymes related with melatonin production in zebrafish embryos in their regular light-dark cycle (14 hours of light/10 hours of darkness) for 72 hours after fertilization. However blue LED exposed zebrafish embryos all died at 72 hpf, therefore, it was not possible to evaluate the effects of blue LED exposure.

In previous studies, blue light in the wavelengths between 400 to 500 nm (near-ultraviolet) have been shown to be the most harmful and damage cells, gametes, and embryos^29,30^. Godley *et al*. reported increased mitochondrial DNA damage in visible light exposed human primary retinal epithelial cells, particularly in blue region spectrum and showed that the superoxide anion was the main reactive oxygen species that caused mitochondrial DNA lesions^30^. Short-wavelength visible blue light has also toxic effects in some insects. Shibuya *et al*. suggested that the growth stage of insects and the wavelength determine the lethal effects of blue light and showed that in *D. Melanogaster* blue light was lethal to all growth stages^31^. Accordingly, in our study, the most prominent increases were observed in lipid peroxidation and nitric oxide levels and the most significant decrease in SOD activity was observed in the white LED light exposed group.

White LED has a broad spectrum. In our study white LED exposure resulted in the loss of yellow pigmentation, decreased body length and locomotor activity, oxidant- antioxidant imbalance and decreased expressions of *aanat2*, *mtnr1ba*, and *pax7*. Histological analysis of this group revealed disorganization of the spaces among photoreceptor cells, decreased the total retinal thickness and photoreceptor cell layer thickness compared with the control group.

In our study, decreased *mtnr1ba* expressions were also observed in the red LED group whereas increased *mtnr1ba* expressions were found in the green and orange LED groups. On the other hand, *aanat2* expressions decreased in all LED light groups with the most dramatic decrease being in the white LED group. As melatonin acts as an antioxidant, decreased *aanat2* expressions in all LED light exposed groups may be related with increased reactive oxygen species as evidenced by increased LPO in all LED light exposed groups. On the other hand the most marked increases were observed in the red and white LED groups which may be associated with decreased *mtnr1ba* expressions in these groups. Choi *et al*. used brain cells of Goldfish to investigate the effects of red (peak at 630 nm), green (peak at 530 nm) and blue (peak at 450 nm) LED lights and white fluorescent light as the control group on melatonin receptor 1 (MT1) mRNA and melatonin levels^32^. Different from our results, they found decreased MT1 mRNA expression and melatonin levels in the green and blue LED treatment groups. On the other hand, both melatonin and MT1 mRNA levels in white fluorescent and red LED light groups were significantly higher than the green and blue LED treated groups^32^. In the same study, when the samples were collected at night-time (03:00), significantly increased MT1 mRNA expression and plasma melatonin levels were reported than the samples collected during day-time^32^. Similar to the results of Choi *et al*., Shin *et al*. found increased melatonin levels in fluorescent and red LED light exposed groups when compared to green and blue LED groups^33^. The differences of our study from these studies are, higher wavelengths of the red (650–670 nm) and blue (455–475 nm) LED lights, the sample collection at day-time and use of zebrafish embryo.

Decreased body length and locomotor activity in the white LED exposed embryos were evident in the white LED exposed embryos when compared with the white light control and other exposure groups. This inhibition may be suggested to be due to the high blue light spectrum in white LED as blue light has been reported to inhibit the development of growth stages of different species^28–31,34^.

Villamizar *et al*. reported that constant white light exposure led to the diminishment of the growth rate at 5 dph zebrafish larvae and the retardation of growth remained until the end of their experiment^34^. Our study is different from these studies as zebrafish embryos were exposed to LED light sources during 14 hours of daytime. When compared with the compact fluorescent lamp conventionally used as the source of white light, blue-rich LED white light has been shown to induce retinal photochemical injury (RPI) with a higher potential^35^. In our study, decreased *aanat2* and *mtnr1ba* expressions suggest suppression of melatonin production was most evident in the white LED light exposed group. As melatonin production is wavelength dependent, short wavelengths have been reported to be very effective in suppressing melatonin production^24,36^. Accordingly Falchi *et al*. suggested that exposure to white light LEDs suppressed body’s production of melatonin more than other lights^36^.

Danilova *et al*. reported that melatonin-treated zebrafish embryos developed faster and suggested that MT2, homologous to zebrafish *mtnr1ba*, played the major role in the acceleration of development^13^. They suggested that with the onset of endogenous melatonin production and melatonin receptor expression, melatonin increases cell proliferation in the embryo and stimulates the development of zebrafish^13^.

As an important free radical scavenger melatonin, enhances the antioxidant potential of cells by the induction of antioxidant enzyme synthesis such as SOD, and increasing glutathione levels^37^. Therefore the oxidant-antioxidant imbalance in the white LED light exposed embryos may be suggested to be the result of melatonin suppression. The contribution of ROS to damage caused by blue light irradiation has been shown in other studies^37,38^. Kuse *et al*. showed that blue light LED irradiation generated reactive oxygen on mouse cultured retinal cells^38^. In another study, blue light LED irradiation induced oxidation of phospholipids in mouse retina^39^. Accordingly, in our current study, impaired oxidant antioxidant balance in white LED light exposed group is consistent with these finding as white LED light has high blue spectrum content. In the green LED exposed group, the increase in LPO levels was less than other exposure groups which might be due to the high SOD activity in the same group. On the other hand, the red LED exposed group had apparent increases in LPO and NO levels and decreases in SOD and GST activities.

Loss of yellow pigmentation in white light exposed embryos is an important finding of our study. In order to evaluate the underlying mechanism *pax7* expression was evaluated and decreased *pax7* expression was evident in orange, red and white light exposed groups. White light exposed group had the most dramatically decreased *pax7* expression which may be related to the loss of yellow pigmentation in this group as Pax7 regulates xanthophore pigmentation^16^. In order to understand the mechanism underlying the inhibitory effect of white LED light on xanthophore pigmentation, we have investigated *crestin* gene expression by *in situ* hybridization. *crestin* gene which is expressed in early embryogenesis in the neural crest progenitors of zebrafish marks the neural crest during embryonic development and then becomes unobservable at 72 hpf^40^. Chromatophores of zebrafish are originated from neural crest cells in the embryo/early larvae and crestin has been reported to go ahead of Pax7 in the premigratory crestin^41^. In our study, white LED light exposure decreased crestin expression at 48 hpf which may have led to decreased *pax7b* expressions at 72 hpf.

By interfering with human the circadian system artificial lighting has the potential to become an important risk factor for different diseases including cancer, especially for industrialized countries. Accordingly, our study showed that white light exposure decreased the expressions of genes related to melatonin production and xanthophore pigmentation, disrupted the oxidant-antioxidant status and embryonic development of the eye in zebrafish embryos. We believe that more studies should be performed and the necessary regulations should be made regarding this risk factor.

Our study has some limitations. Although we measured *aanat2*, melatonin-expressing gene, and *mtnr1ba*, melatonin receptor-expressing gene we did not measure melatonin levels directly. Moreover performing these analyses with dose-dependent designs would strengthen our findings.

## Methods

### Maintenance of zebrafish

Wild type AB/AB Strain zebrafish were maintained in apparently disease-free conditions. Animals were kept in an aquarium rack system (Zebtec, Tecniplast, Italy) at 27 ± 1 °C under a light/dark cycle of 14/10 h. Animals were fed twice a day with commercial flake fish food complemented with live Artemia. All experiments were performed using reverse osmosis water supplemented with 0.018 mg L^−1^ Instant Ocean™ salt^42^. Since the embryos used were no older than 5 days old, Council of Europe (1986), Directive 86/609/EEC or the Marmara University ethics committee did not require license.

### Experimental design

Healthy zebrafish embryos at 4 h post-fertilization (hpf) were divided into 6 groups (40 embryos/6 cm petri dishes) in petri containing 10 mL E3 medium. Every 24 h solutions were replaced and dead embryos were discarded.

Our study focused on the effects of overexposure to light in the visible region, therefore high power LEDs with high LUX values were used for the exposure groups and 400 lux (3200 K) white light was used for the white light control group.

**Figure 9:**
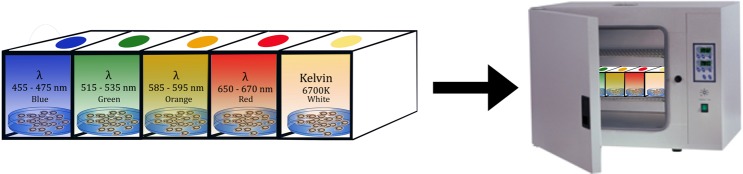
Experimental design. High power LEDs at 1 watt power as blue (455–475 nm), green (515–535), orange (585–595 nm), deep red (650–670 nm), and white (7200 K) were positioned on the petri dishes emitting light at 120^0^ (14 hours of bright light/10 hours of darkness). Each test group was kept in the incubator at 27 ± 1 °C for up to 72 hours under their own light conditions.

**Figure 10:**
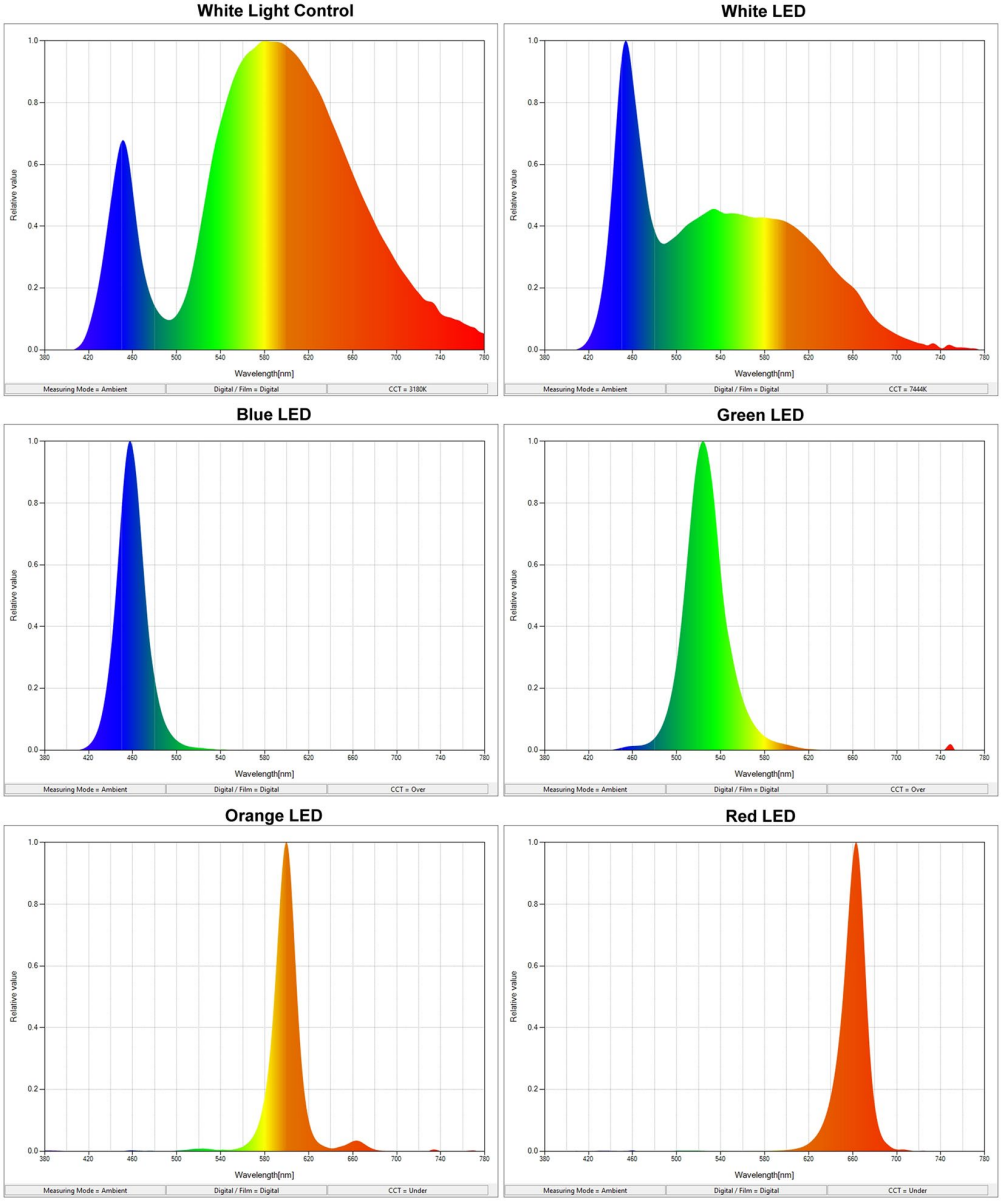
The spectrums of the white light control and LED light groups.The range of light wavelengths that they emit, measured in nanometres are given for the groups.

The high power LEDs at 1-watt power as blue (455–475 nm), green (515–535 nm), orange (585–595 nm), deep red (650–670 nm), and white (7200 K) were positioned on the petri dishes emitting light at 120^0^ (Fig. 9). The spectrums of the white light control and white LED are given in Fig. 10. The lights were set to open at 09:00 and to close at 23:00 (14 hours of bright light/10 hours of darkness) with the self-timer. The photon irradiance of all lamps was adjusted to 1 W/m2. Each test group was kept in the incubator at 27 ± 1 °C for up to 72 hours under their own light conditions. During 72 hpf, the embryos were examined under a stereomicroscope (ZEISS Discovery V8,Germany) to observe morphological abnormalities. The mortality and hatching rates of the groups were recorded daily. The body length measurements of zebrafish embryos at 72 hpf performed from the tip of the nose (tip of the snout) to the base of the caudal fin using the Zeiss software.

### Quantification of pigmentation

A region of interest to measure the pigmentation area was selected and the density of the pigmented area in the white LED light treated embryos was normalized to that in the white light control embryos using Image J software^43^.

### Behavioral analysis

Locomotor activity in vertebrates, including swimming, relies upon neural networks in the brain and spinal cord^44,45^. The first movements are observed in the embryos precisely at 17 hpf and consisted of alternating side-to-side contractions of the tail, much like a metronome. At 72 hpf touch-evoked movement test was performed as described previously^45,46^.

### Biochemical assays

For the biochemical analyses, zebrafish embryos at 72 hpf were used. They were prepared as replicate pools of 72 hpf zebrafish (n = 5, 100 individuals per pool). For each pool 100 embryos were homogenized in 1 ml PBS, followed by centrifuging briefly. The supernatant was used for the determination of biochemical parameters^45^.

### Lipid peroxidation determination

The method of Yagi was used to determine malondialdehyde (MDA) level, an end product of lipid peroxidation (LPO), as thiobarbituric acid reactive substances^45,47^. The extinction coefficient of 1.56 × 10^5^ M^−1^cm^−1^ was used and LPO was expressed in terms of MDA equivalents as nmol MDA/mg protein^45^.

### Nitric oxide determination

Nitric oxide (NO) was determined by the method of Miranda which is based on reducing nitrate to nitrite by vanadium (III) chloride^45,48^. In an acidic media, nitrite and sulfonylamide reacts with N-(1-Naphtyl) ethylenediamine dihydrochloride and complex diazonium compound was formed. The colored complex was measured at 540 nm by a spectrophotometer and results were expressed as nmol NO/mg protein^45^.

### Glutathione-S-transferase determination

The activity of glutathione-s-transferase (GST) was determined based on the spectrophotometric evaluation of the absorbance at 340 nm of the product formed by GSH and 1-chloro-2,4-dinitro-benzenin (CDNB) conjugation^45,49^.

### SOD activity determination

The method based on the ability of SOD to increase the effect of riboflavin-sensitized photo-oxidation of o-dianisidine was used to determine SOD activity in pooled samples. The absorbance of the product was measured in 460 nm by a spectrophotometer. The net absorbance was calculated by measuring absorbances at 0 and 8^th^ minutes of illumination. The results were expressed as U/mg protein^42,50^.

### Reverse transcription (cDNA synthesis) and quantitative real-time PCR

RNA was isolated from the embryos using Rneasy Mini Kit and Qiacube (Qiagen) according to the manufacturer’s instructions. Single-stranded cDNA was synthesized from 1 μg of total RNA using RT^2^ Profiler PCR Arrays (Qiagen). PCRs were performed using the DNA Master SYBR Green kit (Qiagen). PCRs were performed using the DNA Master SYBR Green kit (Qiagen)^51^. The expression of *aanat2* (forward primer 5′-3′: AGGACGCCATCAGTGTGTTT; reverse primer 5′-3′: CTGGCCCAGGAAAACAAGTA), *mtnr1ba* (forward primer 5′-3′ CATTGGTCCCTGATTGGCTG; reverse primer 5′-3′ GTCCCGCCTTTTGATGTCTC) and *pax7b* (forward primer 5′-3′: GGAACAGTACCGCGAATGAT; reverse primer 5′-3′: GAATACACCGCCAAGCTGAT) were evaluated by quantitative RT-PCR using the Qiagen Rotor Gene-Q Light Cycler instrument. β-actin (forward primer: 5′-GCTCACCATGGATGATGATATCGC-3′reverse primer: 5′-GGAGGAGCAATGATCTTGATCTTC-3′) was used as the housekeeping gene. Relative transcript levels were calculated using the DDCT method by normalizing the values to the housekeeping gene^52,53^.

### *In situ* hybridization

*In situ* hybridization was performed on 48 hpf embryos according to the method of Thisse using crestin as the probe^54^.

### Histological evaluations

For the light microscopic investigation, zebrafish embryos were fixed in 10% neutral buffered formalin, dehydrated with alcohol series, cleared with toluene and finally embedded in paraffin for sectioning. After embedding in paraffin, approximately 3 µm thick horizontal serial sections were taken along the nasal–temporal axis by rotary microtome and stained with hematoxylin and eosin (HE). Six random sections of each embryo were selected for retinal morphometric measurement. In each section, the thickness of the total retina and photoreceptor cell layer (PCL) at four different points were measured using the ImageJ 1.46r software (Wayne Rasband National Institutes of Heath, USA). Data obtained from six sections were averaged for each eye. All stained sections were examined and photographed using a digital camera (Olympus DP72, Tokyo, Japan) which was attached to a photomicroscope (Olympus BX51, Tokyo, Japan).

### Statistical analysis

The data were expressed as mean ± SD. Statistical analysis was performed with the use of Graph Pad 7.0 (GraphPad Software, San Diego, CA, USA). Anova test followed by Tukey’s multiple comparisons test was used to analyze the data. P value < 0.05 was considered to be statistically significant.

## Data Availability

The datasets generated and analysed during the current study are available from the corresponding author on reasonable request.
